# Evaluation of medication adherence over a decade following kidney transplantation: integration of biological and self-reported data

**DOI:** 10.1186/s12882-026-05093-8

**Published:** 2026-06-06

**Authors:** Yaprak Sarıgöl Ordin, Serap Arıcan, Büşra Selma Saha, Hale Turhan Damar

**Affiliations:** 1 Surgical Nursing Department, Faculty of Nursing, Dokuz Eylul University, Izmir, Türkiye; 2 Department of Health Care Services, Izmir City Hospital, Izmir, Türkiye; 3 Surgical Nursing Department, Institute of Health Sciences, Dokuz Eylul University, Izmir, Türkiye; 4 Department of Nursing, Faculty of Health Science, İzmir Democracy University, Izmir, Türkiye

**Keywords:** Biological monitoring, Follow-up studies, Kidney transplantation, Immunosuppressive agents, Medication adherence, Self report

## Abstract

**Background:**

This study aims to determine the course of immunosuppressive medication adherence (MA) in individuals who have undergone kidney transplantation for over a decade and to identify the demographic and clinical factors affecting this adherence.

**Methods:**

This retrospective and cross-sectional descriptive study evaluated the biological adherence of 103 kidney transplant recipients (KTRs) at 12 different time points using tacrolimus and cyclosporine blood levels. Self-reported adherence was assessed via The Immunosuppressant Therapy Adherence Scale. Associations between adherence and demographic and clinical variables were also analyzed.

**Results:**

The mean time since transplantation was 11.77 ± 1.46 years. Biologically, 43.3% of patients had low MA. In contrast, self-reported adherence was 82.52%. No statistically significant correlation was found between biological and self-reported adherence outcomes. Patients with low biological adherence presented increased blood urea nitrogen (BUN) levels at 6 months and 9 years post-transplant. Similarly, patients with low self-reported adherence had increased BUN levels at 10 years. Self-reported adherence scores were significantly lower among patients with a history of graft rejection (*p* < 0.001). No significant associations were found between MA and variables such as sex, donor type, or age (*p* > 0.05).

**Conclusions:**

This study identifies significant discrepancies between biological and self-reported methods for evaluating long-term adherence to immunosuppressive therapy. The results indicate that adherence fluctuates over time, highlighting the need for multidimensional assessment strategies. The implementation of standardized, and complementary tools is crucial to improving graft and patient survival. Future multicenter research should investigate pre-transplant predictors to optimize long-term medication adherence.

## Background

Sufficient immunosuppression is essential for both short- and long-term survival following organ transplantation. Accordingly, adherence to immunosuppressive therapy is critical for preventing organ rejection, graft loss, and posttransplant mortality [1–3]. Nonadherence to immunosuppressive medication has been linked to approximately 16% of early graft losses and 20% of antibody-mediated rejections [4]. The prevalence of medication adherence (MA) among kidney transplant recipients (KTRs) varies widely, with a systematic review reporting rates between 36% and 55% [5], whereas a scoping review revealed an even broader range, from 2% to 89% [6]. Several factors have been associated with nonadherence, including younger age, male sex, low social support, unemployment, lower educational attainment, longer time since transplantation, sirolimus-based therapy, living donor transplantation, high chronic disease burden, polypharmacy, and a history of depression [7, 8]. Early identification of recipients with these risk factors and the implementation of close, individualized monitoring are critical strategies for improving adherence and ensuring long-term graft survival.

Regular assessment of treatment adherence is necessary to ensure that immunosuppressive therapy can be continued safely and effectively. The most commonly used direct method in clinical practice is the measurement of therapeutic drug levels; however, this method reflects only short-term adherence. In this context, the term *biological adherence* refers to the objective assessment of medication-taking behavior based on measured blood concentrations of immunosuppressive drugs. Indirect methods include patient self-assessment forms, patient diaries, prescription refill records, and electronic medication monitoring technologies [9]. Among *self-reported measures*, the Immunosuppressant Therapy Adherence Scale (ITAS) is a four-item instrument specifically developed for transplant recipients to assess missed-dose behavior over the previous three months [10]. Each item evaluates the frequency of nonadherent behavior, with higher total scores indicating better medication adherence. A single adherence assessment method is not sufficient for accurately measuring adherence behavior. Therefore, combining multiple methods and repeated assessments ensures more accurate and reliable results [9, 11, 12]. In addition, MA is a dynamic process that may vary over time after transplantation [13]. The literature presents conflicting findings on this issue: some studies have shown that adherence decreases over the long term [14], whereas others have shown that early adherence behavior continues in later periods [15]. On the other hand, some findings report an increase in adaptation between the 3rd and 5th years [16]. These differences highlight the need for an assessment approach that spans time.

Kidney transplantation is a highly effective treatment modality that significantly enhances long-term survival in patients with end-stage renal disease. As the number of transplant recipients continues to rise worldwide with more than 110.000 kidney transplants performed globally in 2024, evaluating long-term MA and identifying the factors that influence it have become increasingly important [17]. Immunosuppressive therapy is essential for maintaining graft function and preventing rejection, yet adherence to these medications can decline over time due to various demographic, clinical, and psychosocial factors. In this context, the present study aims to evaluate long-term immunosuppressive MA among individuals who have been living with a kidney transplant for 10 years or longer by using therapeutic drug levels as an indicator of biological adherence, assessing self-reported adherence measures, and identifying the demographic and clinical factors influencing medication adherence.

## Methods

### Study design and patient population

This study was conducted with a retrospective, cross-sectional, descriptive research design. This study was conducted in the kidney transplantation unit of a training and research hospital. The data were collected between June 2023 and July 2024. The study sample consisted of KTRs who had undergone transplantation at least 10 years prior. Kidney transplant recipients aged 18 years or older, with an ability to speak and understand Turkish language were included. All participants provided written informed consent prior to data collection. The study was reviewed and approved by the Dokuz Eylul University Non-Invasive Research Ethics Committee (Approval No: 2023/17 − 08; May 24, 2023).

**Fig. 1:**
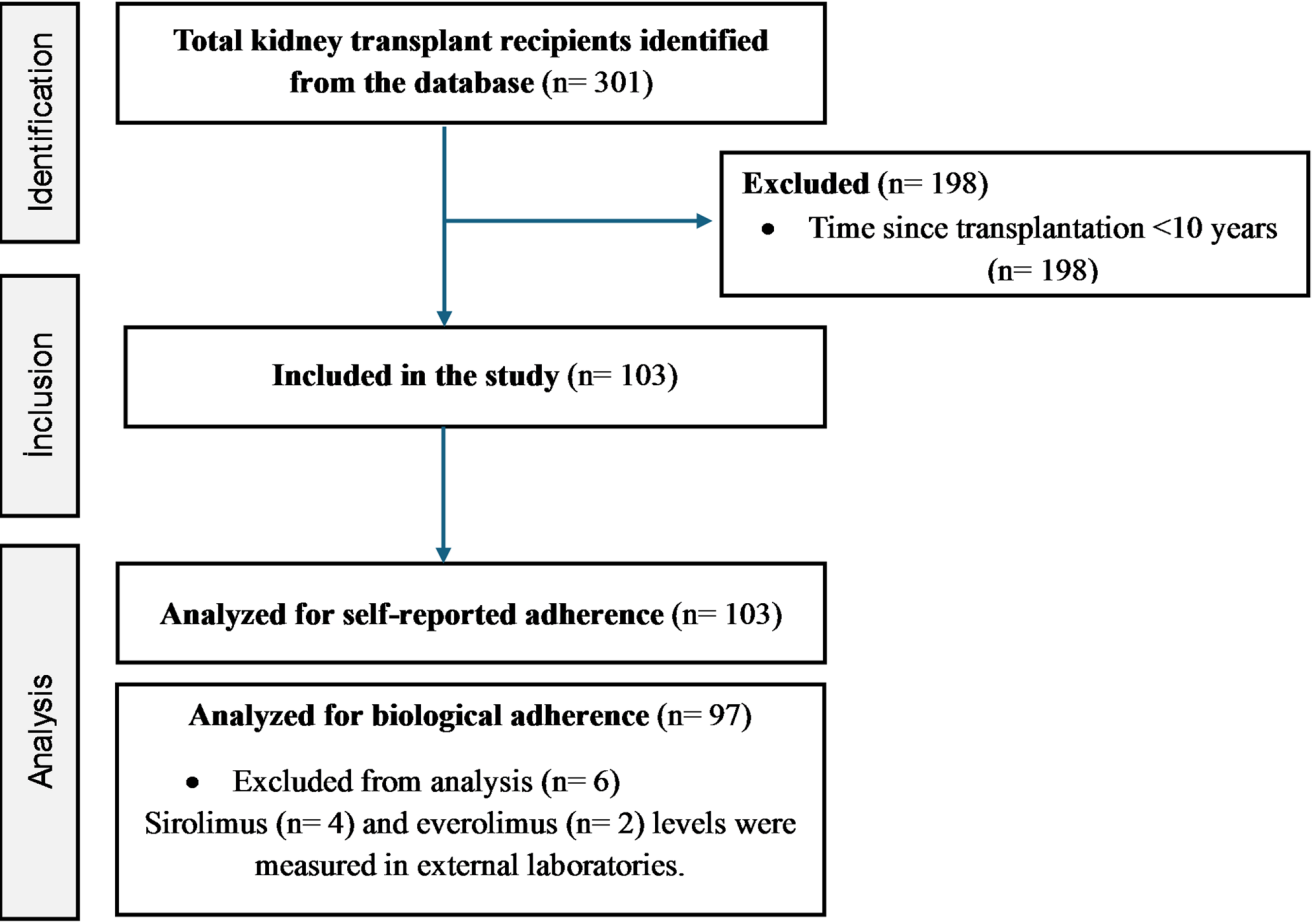
Study flow diagram of the retrospective cohort

At the time of data collection, 301 KTRs were under follow-up. Among them, 103 patients had undergone kidney transplantation at least 10 years earlier and met the inclusion criteria. The study flow diagram is shown in Fig. 1. Following the independent samples t test, a post hoc power analysis was conducted via G*Power 3.1 to assess the adequacy of the sample size. The analysis revealed that, for the sample size of 103 participants, a significance level of α = 0.05, and an effect size of d = 0.380, the achieved statistical power (1–β) was 0.98. This finding indicates that the sample size was highly sufficient for detecting the observed effect.

### Research questions

1. Is there a significant relationship between biological adherence, assessed through therapeutic drug levels, and self-reported MA in long-term KTRs?
2. Do demographic and clinical characteristics-such as sex, age at transplantation, type of underlying comorbidity, donor type (living or deceased), and the donor-recipient relationship, significantly influence biological adherence and self-reported medication adherence?
3. How do immunosuppressive drug levels vary across post-transplantation periods in KTRs, and what is their relationship with biological and self-reported medication adherence?

### Variables and measurements

Self-reports and biological measurements were used to evaluate MA. Biological MA was assessed on the basis of tacrolimus and cyclosporine plasma levels. The blood plasma levels and protocol modifications over the 10-year post-transplant period were retrospectively obtained from patient medical records. According to the kidney transplantation center’s protocol, the therapeutic plasma range for tacrolimus was defined as 10–12 ng/mL during the first 3 months post-transplant and 5–10 ng/mL thereafter. For cyclosporine, the evaluation was based on the C0 concentration. Given that the number of patients with C2 measurements was limited, these data were excluded from the analysis. The therapeutic C0 range for cyclosporine was defined as 200–400 ng/mL between 1 and 3 months, 150–300 ng/mL between 4 and 12 months, and 100–200 ng/mL beyond 12 months.

The 10-year biological MA was evaluated in 97 patients. During the 10-year follow-up period were assessed at 12 measurement points: the 3rd month, the 6th month, and the 1st through 10th years post-transplant. For each assessment patients were classified as adherent if their tacrolimus and/or cyclosporine plasma levels fell within the therapeutic range. Conversely, levels outside this range were categorized as non-adherent, as both sub-therapeutic and supra-therapeutic concentrations may indicate irregular medication intake or inadequate dosage management As a result of this evaluation, patients were categorized into five groups on the basis of their MA levels:

- 100% adherent: adherent at all 12 measurement points,
- 75% adherent: adherent at 8–11 measurement points,
- 50% adherent: adherent at 5–7 measurement points,
- 25% adherent: adherent at 1–4 measurement points,
- 0% adherent: not adherent at any measurement point.

Self-reported MA was assessed via ITAS [10]. This study evaluated the MA of patients who had undergone kidney transplantation at least 10 years prior. Self-reported adherence was assessed once at the time of data collection. For example, a patient could have been in their 10th or 17th posttransplant year at the time of assessment; in either case, self-reported adherence was evaluated as a single measurement. ITAS was originally developed by Morisky et al. (1986) to assess adherence to antihypertensive medications among patients with hypertension and was later adapted for organ transplant recipients by Chisholm et al. in 2005 [10]. The ITAS consists of four items assessing the frequency of missed doses of immunosuppressive medication over the previous three months. The items evaluate whether patients: (1) forgot to take their medication, (2) were careless in taking their medication, (3) stopped taking their medication when feeling better, and (4) stopped taking their medication when feeling worse. Responses to each item are categorized as 0%, 1–20%, 21–50%, or > 50% nonadherence and scored from 3 to 0 points, respectively. Accordingly, a score of 3 indicates no missed doses in the past three months, whereas scores of 2, 1, and 0 correspond to nonadherence rates of 1–20%, 21–50%, and greater than 50%. The total ITAS score ranges from 0 to 12, with higher scores indicating better adherence to immunosuppressive medication. The Turkish validity and reliability study of the scale was conducted by Madran et al. [18]. In this study, patients who received the maximum score of 12 were classified as adherent, whereas those with lower scores were considered nonadherent.

Sociodemographic and clinical characteristics (age, sex, date of transplantation, donor type and relationship, etiology of kidney disease leading to transplantation, presence of comorbid chronic disease, preemptive transplantation, retransplantation, and rejection history) were obtained from medical records. Regarding comorbidities, participants were first asked to indicate the presence of any chronic disease, followed by an open-ended question to specify the diagnosis. Additionally, patients’ blood urea nitrogen (BUN) and creatinine levels were evaluated at a total of 12 time points: 3 months, 6 months, and annually from year 1 to year 10.

### Data analysis

Statistical analyses were performed via the IBM Statistical Package for the Social Sciences (SPSS) version 29.0 (IBM Corp., Armonk, NY, USA). Descriptive characteristics were evaluated via frequencies, percentages, means, and standard deviations. The Kolmogorov–Smirnov test was applied to assess the normality of the data distribution. Changes in biological MA over time were analyzed via Cochran’s Q test. The relationship between biological and self-reported MA was assessed via the chi-square test. The associations between MA and tacrolimus levels and biochemical parameters such as BUN and creatinine were evaluated via the Mann–Whitney U test. Similarly, the relationships between self-reported MA and BUN and creatinine levels were analyzed via the Mann–Whitney U test. The relationships between MA and variables such as sex, age at transplantation, type of comorbidity, donor type, donor relationship, and history of rejection were analyzed via chi-square, Mann–Whitney U, or Kruskal–Wallis tests, depending on the level of measurement of the variables.

## Results

A total of 103 patients were included in the study. The mean age of the patients was 52.43 ± 11.76 years (min-max = 23–77). When categorized into age groups, 12.6% (*n* = 13) patients were aged 18–39 years, 55.3% (*n* = 57) were aged 40–59 years, and 32.0% (*n* = 33) were aged ≥ 60 years. The mean time elapsed since transplantation was 11.77 ± 1.46 years (min-max = 10–20). Among the patients, 62.1% (*n* = 64) were male. At least one comorbidity was presented in 76.7% (*n* = 79) of the patients, with hypertension being the most prevalent 76.7% (*n* = 73). One patient had undergone preemptive transplantation, and five patients (4.9%) were retransplant recipients. The etiology of kidney disease leading to transplantation was unknown in 24.3% (*n* = 25) of the patients. The deceased donation rate was 52.43% (*n* = 53). In addition, 10.7% (*n* = 11) developed rejection, and the mean time to rejection was 5 ± 4.63 years.

### Medication adherence

#### Biological medication adherence

**Fig. 2:**
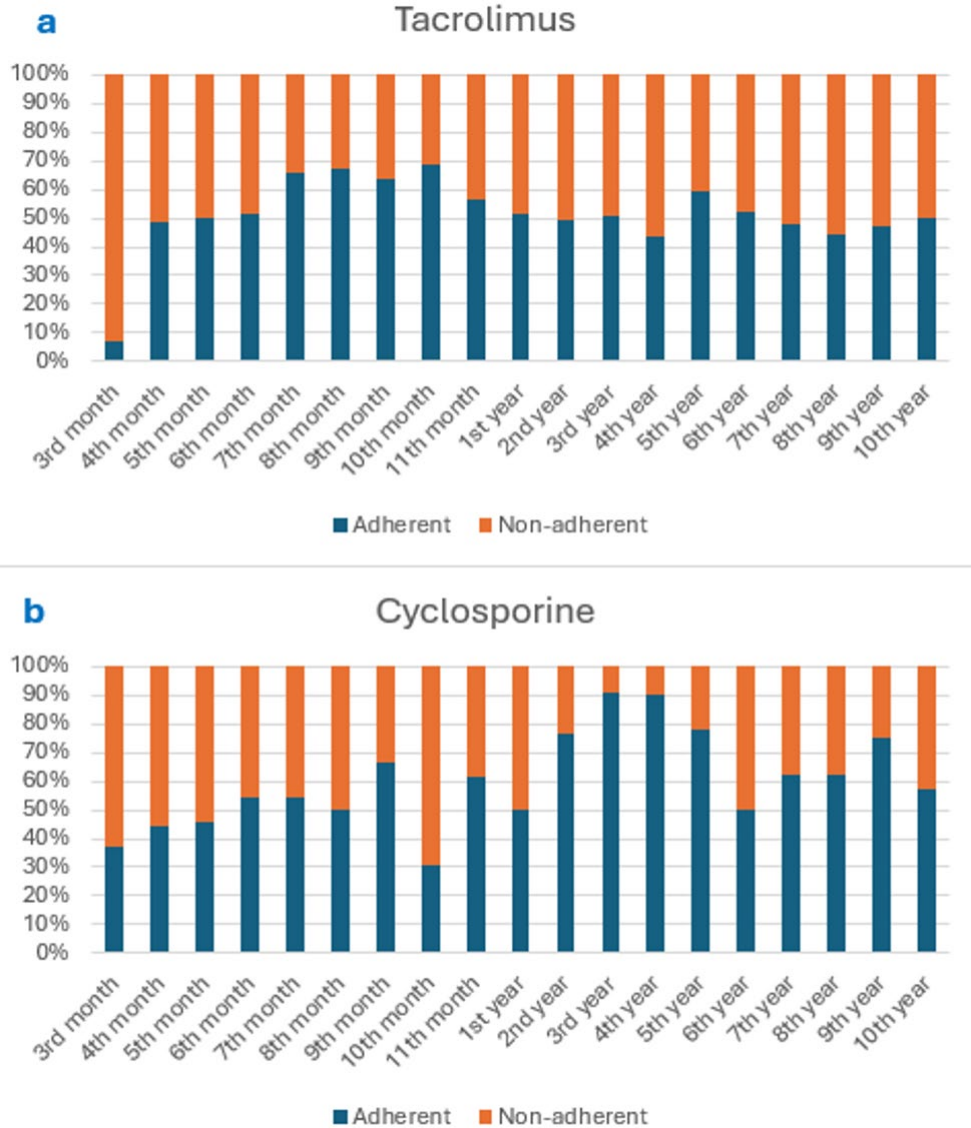
Longitudinal distribution of biological adherence from the 3rd month to the 10th year post-transplantation among kidney transplant recipients. (**a**) Tacrolimus and (**b**) Cyclosporine

All measured tacrolimus and cyclosporine plasma levels were categorized according to adherence status. The distribution of patients’ 10-year MA, based on tacrolimus and cyclosporine plasma levels, is shown in Fig. 2. When the MA levels were categorized according to the 12 measurements obtained over the 10-year period, 1 patient (1.03%) was 100% adherent; 30 patients (30.93%) were 75% adherent; 24 patients (24.74%) were 50% adherent; 37 patients (38.15%) were 25% adherent; and 5 patients (5.16%) were 0% adherent. Patients demonstrating 0% or 25% adherence were grouped as having low adherence, representing 43.3% of the total sample (*n* = 42). A significant difference in tacrolimus adherence over time was observed over the 10-year follow-up period after kidney transplantation, specifically between the 3rd month, 6th month, and 10th year (Cochran’s Q = 70.871, *p* < 0.001). Post-hoc pairwise comparisons with Bonferroni correction revealed that medication adherence at the 3rd month was significantly different from all other time points, including the 6th month and the 10th year (*p* < 0.001).

#### Self-reported medication adherence

The mean ITAS score was 11.54 ± 1.15 (min–max = 7–12, *n* = 103). Patients who received the maximum score on the ITAS were classified as adherent, resulting in an overall adherence rate of 82.52% (*n* = 85).

### Relationship between biological and self-reported medication adherence

No statistically significant relationship was found between the 10-year biological assessment results and self-reported medication adherence among KTRs (χ²=0.002, *p* = 0.965).

### Relationship between biological medication adherence and the BUN-creatinine ratio

**Fig. 3:**
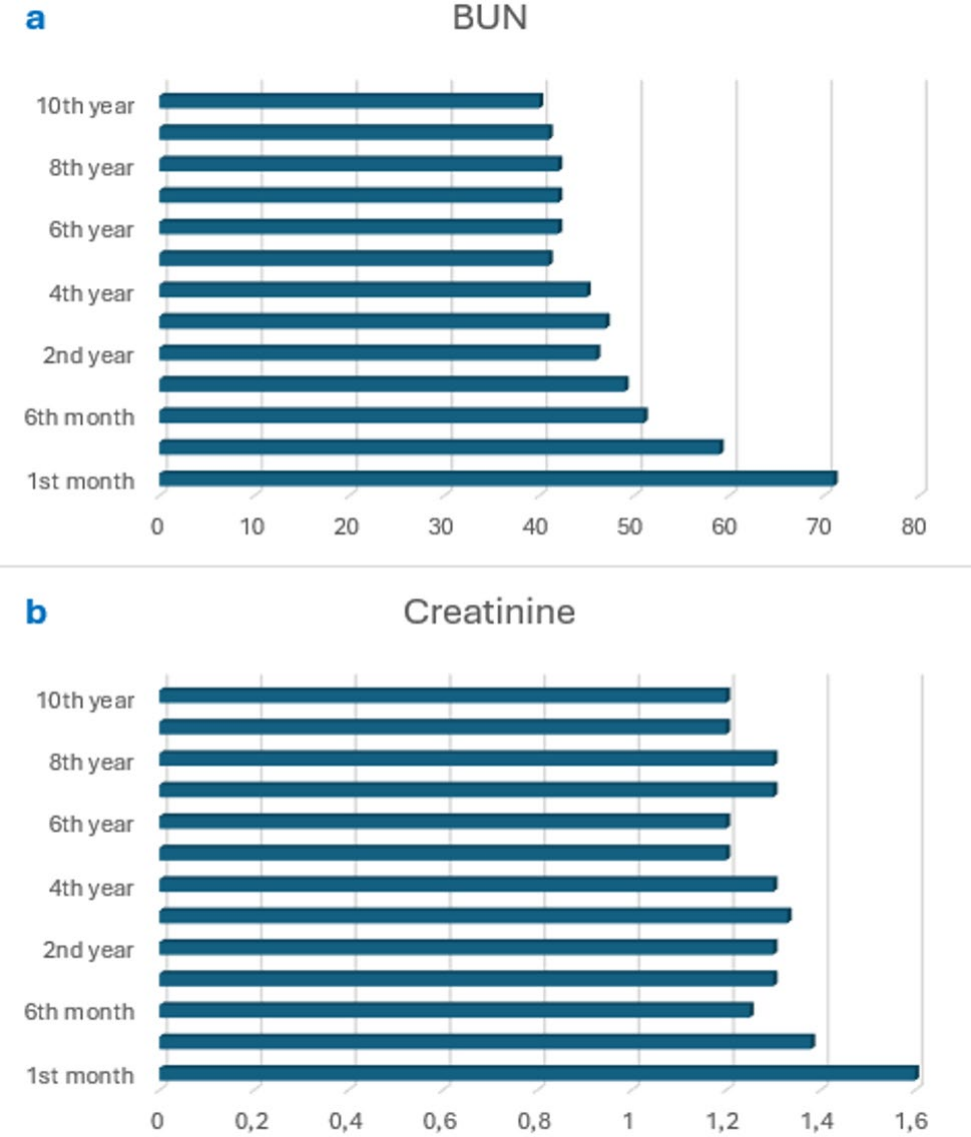
Longitudinal changes in renal function parameters from the 1st month to the 10th year post-transplantation among kidney transplant recipients. (**a**) Blood Urea Nitrogen (BUN) (**b**) Creatinine

The 10-year distribution of patients’ BUN and creatinine levels are presented in Fig. 3. Differences in BUN and creatinine levels according to biological MA status are shown in Table 1. Among patients receiving tacrolimus, the nonadherent group had a significantly higher mean BUN level at the 6th month (74.81 ± 44.21) than the adherent group (52.39 ± 28.34) (U = 462.00, *p* = 0.016). Among patients receiving cyclosporine, the nonadherent group also had a higher mean BUN level at the 9th year (1.17 ± 0.25) than the adherent group (0.89 ± 0.19) (U = 0.00, *p* = 0.046). A statistically significant negative correlation was found between self-reported MA and BUN levels at 10-year follow-up (U = 329.50, *p* = 0.001). However, no significant association was found between self-reported MA and creatinine levels (U = 755.50, *p* = 0.934) (Table 2).

**Table 1:** Effect of biological medication adherence on BUN and creatinine levels in kidney transplant recipients

Time	Tacrolimus		Cyclosporine	
	BUN	Creatinine	BUN	Creatinine
3rd month	U = 166.50 *p* = 0.897	U = 148.50 *p* = 0.605	U = 3.00 *p* = 0.121	U = 2.00 *p* = 0.071
6th month	U = 462.00 ***p*** = 0.016*	U = 662.50 *p* = 0.816	U = 12.00 *p* = 0.917	U = 8.00 *p* = 0.347
1st year	U = 625.00 *p* = 0.316	U = 687.00 *p* = 0.719	U = 9.00 *p* = 0.150	U = 6.00 *p* = 0.053
2nd year	U = 699.00 *p* = 0.669	U = 734.50 *p* = 0.947	U = 13.00 *p* = 0.735	U = 11.50 *p* = 0.553
3rd year	U = 644.00 *p* = 0.661	U = 576.00 *p* = 0.176	U = 2.00 *p* = 0.343	U = 1.00 *p* = 0.205
4th year	U = 693.00 *p* = 0.863	U = 588.50 *p* = 0.204	U = 5.00 *p* = 1.000	U = 2.00 *p* = 0.337
5th year	U = 731.50 *p* = 0.838	U = 640.00 *p* = 0.262	U = 6.00 *p* = 0.770	U = 4.00 *p* = 0.372
6th year	U = 737.50 *p* = 0.348	U = 639.00 *p* = 0.063	U = 4.50 *p* = 0.309	U = 5.50 *p* = 0.468
7th year	U = 855.50 *p* = 0.826	U = 846.00 *p* = 0.760	U = 1.00 *p* = 0.53	U = 2.00 *p* = 0.101
8th year	U = 762.50 *p* = 0.417	U = 829.50 *p* = 0.844	U = 2.50 *p* = 0.134	U = 6.00 *p* = 0.651
9th year	U = 876.50 *p* = 0.836	U = 874.50 *p* = 0.822	U = 0.00 ***p*** = 0.046*	U = 0.50 *p* = 0.064
10th year	U = 887.00 *p* = 0.746	U = 917.00 *p* = 0.948	U = 5.50 *p* = 0.858	U = 6.00 *p* = 1.00

*p < 0.05, Mann-Whitney U, BUN: blood urea nitrogen

**Table 2:** Effect of self-reported medication adherence on BUN and creatinine levels in kidney transplant recipients

Parameters	Adherent (ITAS = 12) Median (Min–Max) *n* = 85	Non-adherent (ITAS ≤ 11) Median (Min–Max) *n* = 18	
BUN (mg/dL) (*n* = 103)	38.00 (18–102)	62.95 (24–141)	U = 329.50 ***p*** = 0.001*
Creatinine (mg/dL) (*n* = 103)	1.20 (0.60–5.56)	1.15 (0.70-6.00)	U = 755.50 *p* = 0.934

*p < 0.05, Mann-Whitney U, BUN: blood urea nitrogen

### Factors associated with medication adherence

In the analysis of factors affecting MA, no statistically significant relationships were found between sex, age at transplantation, type of comorbidity, donor type, or donor relationship and tacrolimus plasma levels at the 3rd month, 6th month, 1st year, 5th year, or 10th year (*p* > 0.05). No statistically significant association was found between age groups and self-reported medication adherence (χ²=5.048, *p* = 0.080). A statistically significant association was observed between sex and tacrolimus plasma level at the 7th year (χ²=4.582, *p* = 0.032), with female patients exhibiting higher tacrolimus concentrations (U = 648.00, *p* = 0.033). With respect to self-reported MA, no statistically significant relationships were found with age (*r* = 0.107, *p* = 0.28), sex (U = 1201.00, *p* = 0.63), type of comorbidity (KW = 5.949, *p* = 0.65), donor type (U = 1313.50, *p* = 0.91), or donor relationship (KW = 4.007, *p* = 0.64). Patients who experienced rejection had lower mean self-reported adherence scores (9.73 ± 2.28) than did those without rejection (11.77 ± 0.66) (U = 225.50, *p* < 0.001).

## Discussion

This study examined immunosuppressive MA and the factors influencing it over a decade among KTRs via both biological and self-reported assessment methods. MA plays a critical role in graft survival and overall patient prognosis [19]. Therefore, evaluating adherence behaviors is particularly important in the long-term management of transplant recipients.

In the present study, biological assessment revealed that 43.3% of patients demonstrated low MA, and only one patient achieved full adherence. These rates approach the upper limits of the wide range of nonadherence rates reported in the literature [5, 6]. The exceptionally low rate of full adherence highlights the challenge of maintaining long-term sustainability to immunosuppressive therapy. Previous studies have similarly reported an association between nonadherence and lower tacrolimus levels [20, 21]. Accordingly, monitoring therapeutic blood levels and adherence to prescribed regimens should be integrated into routine transplant care.

In this study, fluctuations in biological adherence over time were observed on the basis of both tacrolimus and cyclosporine plasma levels. This finding is consistent with the variable results reported in the literature. While some studies have shown that the time elapsed since transplantation does not influence MA [21, 22], others have demonstrated that adherence may decrease or, in some cases, increase during certain post-transplant periods [5, 7, 8, 14]. This variability may arise not only from temporal factors but also from multidimensional effects such as treatment fatigue, psychological exhaustion, financial burden, and individual risk profiles [16, 23]. Therefore, MA should be evaluated not only in the early posttransplant stages but also as a long-term process that evolves [6].

In this study, the self-reported MA rate was relatively high at 82.52%. However, no statistically significant associations were detected between self-reported and biologically measured adherence. This inconsistency has been frequently reported in the literature and is largely attributable to the inherent differences between subjective and objective assessment methods [24]. Self-report questionnaires such as the ITAS primarily reflect patients’ medication-taking behavior within a limited time frame and may therefore be insufficient for evaluating long-term medication adherence. Furthermore, these instruments are susceptible to social desirability bias, leading patients to overreport their adherence levels [25, 26]. Similarly, therapeutic plasma levels are influenced not only by patient behavior but also by drug–drug or drug–food interactions, as well as interindividual metabolic differences [24]. Therefore, considering the inherent limitations of both approaches, their combined use at multiple time points would yield a more comprehensive and reliable assessment of MA.

The impact of medication nonadherence on clinical biomarkers was evaluated using both biological and self-reported adherence measures. Increased BUN levels were observed at the 6th month and 9th year among biologically nonadherent patients and at the 10th year among self-reported nonadherent patients; however, no significant association was found with creatinine levels. Isolated elevations in BUN without concomitant increases in creatinine may not reliably reflect true graft dysfunction, as BUN can be influenced by non-renal factors such as hydration status, protein metabolism, dietary protein intake, and catabolic state [27]. Previous research has indicated that creatinine levels increase only in advanced allograft damage and have limited sensitivity in detecting early renal damage [28]. In the absence of graft biopsy confirmation, observed biomarker changes cannot be considered definitive evidence of structural graft damage and should instead be evaluated as potential early warning signals. Accordingly, BUN and creatinine should be interpreted as complementary indicators, rather than stand-alone markers, in the long-term evaluation of graft function.

In this study, no significant associations were found between adherence and sex, age, type of comorbidity, donor type, or donor relationship. This finding is consistent with previous studies [5, 6, 29]. However, the observation of maintenance within therapeutic range in female patients at the seventh year suggests that sex-related differences may emerge over time. This finding cannot be attributed to a single explanatory factor and may instead reflect the complex interplay of biological, pharmacokinetic, and behavioral mechanisms. Current research also indicates that the association between sex and medication adherence remains inconclusive [29]. The finding that individuals experiencing rejection report lower MA highlights the direct clinical impact of nonadherence. Similarly, higher rejection rates have been reported among KTRs with nonadherence to immunosuppressive therapy [30]. Therefore, adherence should be regularly assessed over time in all patients to identify potential nonadherence issues and implement timely, appropriate interventions [13].

This study has several limitations. First, the data were obtained retrospectively from medical records only, and self-reported adherence was assessed at a single time point. Second, the study was conducted at a single center with a relatively small sample size, which may limit the generalizability of the findings. Third, the use of self-reported measures may be susceptible to social desirability bias, possibly resulting in overestimation of actual adherence levels. Finally, psychosocial variables were not evaluated, and detailed adherence-related factors, such as medication timing, were not assessed.

## Conclusions

This study conducted a comprehensive analysis of long-term immunosuppressive drug adherence by biological and self-reported assessment methods, revealling differences between these measurement approaches. The results indicate that MA fluctuates over time, highlight the necessity of complementary assessment methods, and demonstrate that nonadherence behaviors can directly affect clinical outcomes. Regular assessment of MA through multidimensional approaches in long-term transplant recipients is recommended, as this strategy may effectively improve both graft and patient survival. Future multicenter studies utilizing standardized and multidimensional adherence assessment methods are needed to enhance understanding of the complex nature of adherence. Additionally, investigating medication adherence during the pre-transplant period and its association with post-transplant adherence may help identify early predictors of long-term low medication adherence.

## Data Availability

The datasets generated or analyzed during the current study are available from the corresponding author upon reasonable request.

## Abbreviations

ITAS: Immunosuppressive Therapy Adherence Scale
BUN: Blood urea nitrogen
KTR: Kidney transplant recipient
MA: Medication adherence
